# Expression of *RPRM/rprm* in the Olfactory System of Embryonic Zebrafish (*Danio rerio*)

**DOI:** 10.3389/fnana.2018.00023

**Published:** 2018-03-27

**Authors:** Karen Stanic, Alonso Quiroz, Carmen G. Lemus, Ignacio A. Wichmann, Alejandro H. Corvalán, Gareth I. Owen, Juan C. Opazo, Miguel L. Concha, Julio D. Amigo

**Affiliations:** ^1^Departamento de Fisiología, Facultad de Ciencias Biológicas, Pontificia Universidad Católica de Chile, Santiago, Chile; ^2^Anatomy and Developmental Biology Program, Faculty of Medicine, Institute of Biomedical Sciences, Universidad de Chile, Santiago, Chile; ^3^Advanced Center for Chronic Diseases, Santiago, Chile; ^4^UC Center for Investigation in Oncology, Pontificia Universidad Católica de Chile, Santiago, Chile; ^5^Departamento de Oncología y Hematología, Facultad de Medicina, Pontificia Universidad Católica de Chile, Santiago, Chile; ^6^Millennium Institute on Immunology and Immunotherapy, Santiago, Chile; ^7^Instituto de Ciencias Ambientales y Evolutivas, Facultad de Ciencias, Universidad Austral de Chile, Valdivia, Chile; ^8^Biomedical Neuroscience Institute, Universidad de Chile, Santiago, Chile

**Keywords:** reprimo, zebrafish (*Danio rerio*), olfactory systems, opticum tectum, neurogenesis

## Abstract

The Reprimo (*RPRM*) family is composed of highly conserved single-exon genes. The expression pattern of this gene family has been recently described during zebrafish (*Danio rerio*) embryogenesis, and primarily locates in the nervous system. Its most characterized member, *RPRM*, which duplicated to give rise *rprma* and *rprmb* in the fish lineage, is known to act as a tumor-suppressor gene in mammalian models. Here, we describe in detail the spatiotemporal expression of three *rprm* genes (*rprma, rprmb*, and *rprm*l) within distinct anatomical structures in the developing peripheral and central nervous system. In the zebrafish, *rprma* mRNA is expressed in the olfactory placodes (OP) and olfactory epithelium (OE), *rprmb* is observed in the tectum opticum (TeO) and trigeminal ganglion (Tg), whereas *rprml* is found primarily in the telencephalon (Tel). At protein level, RPRM is present in a subset of cells in the OP, and neurons in the OE, TeO, hindbrain and sensory peripheral structures. Most importantly, the expression of *RPRM* has been conserved between teleosts and mammals. Thus, we provide a reference dataset describing the expression patterns of *RPRM* gene products during zebrafish and mouse development as a first step to approach the physiological role of the *RPRM* gene family.

## Introduction

Reprimo is a family of poorly characterized single-exon genes composed by three paralogs: Reprimo (*RPRM), RPRM-Like (RPRML)*, and *RPRM3* (Wichmann et al., 2016). *RPRM* and *RPRML* have been differentially retained in most vertebrates, including humans, whereas *RPRM3* has been retained only in a fraction of vertebrates, including teleost fish (Wichmann et al., 2016). RPRM is a highly glycosylated cytoplasmic protein that induces cell cycle arrest at G2 in a p53-dependent manner, by inhibiting Cdc2-cyclin B1 complex activity through an as yet unidentified cytoplasmic mechanism (Ohki et al., 2000; Taylor and Stark, 2001). *RPRM* expression is altered in several types of cancers (Sato et al., 2006; Xu et al., 2012; Saavedra et al., 2015), but there is no evidence to date for *RPRM* physiological function.

Recently, we showed that *RPRM*/*rprm* genes are expressed in the zebrafish brain and that the expression domains are conserved between fish and mammals (Figueroa et al., 2017). Since teleost fish underwent an extra round of whole-genome duplication, zebrafish have retained duplicated copies of *RPRM* genes: *rprma* and *rprmb* (Wichmann et al., 2016). Importantly, a precise temporal description of RPRM protein expression during the development of neuronal structures is still missing. Such knowledge is fundamental to advance in our understanding of the possible role of this gene family in neuronal development.

To this end, the zebrafish (*Danio rerio*) is an established model to study early nervous system development, which combines the complexity of a vertebrate organism with the easy-to-use and high-throughput capabilities of *in vitro* models (MacRae and Peterson, 2015). Moreover, zebrafish is very well-suited for systematic analysis of gene expression patterns at transcript level by whole-mount *in situ* hybridization (WISH), and at protein level by immunohistochemistry/immunofluorescence (IHC/IF). Additionally, many of the components that regulate developmental processes are evolutionarily conserved among vertebrate species (Saraiva et al., 2015; Hildebrand et al., 2017).

Herein we examine the temporal and spatial expression patterns of *RPRM* gene-products (mRNA and protein) during neural development. In zebrafish, *rprm* (*rprma, rprmb)* and *rprml* transcripts are expressed in confined regions of the embryonic nervous system including the olfactory placode (OP) and epithelium (OE). Our data constitutes the first evidence that *RPRM* transcripts and proteins are expressed at early stages of OP and OE development. Furthermore, we show that *RPRM* expression in the olfactory system is conserved between zebrafish and mouse. Our study using the zebrafish model system sets the basis to examine the functional role of the *RPRM* gene family in neural development.

## Materials and methods

### Multiple sequence alignment

We annotated *RPRM* genes in human, mouse and zebrafish. Complete amino acid sequences for the three paralogs in the three species were aligned using the L-INS-i strategy from MAFFT v.7 (Katoh and Standley, 2013). Potential domains were predicted using the TMHMM method (http://www.cbs.dtu.dk/services/TMHMM/) as implemented in Geneius Software. To assess the potential cross-reactivity between human RPRM antibody and zebrafish Rprma/Rprmb proteins, the immunogenic sequence from human RPRM used to generate rabbit polyclonal anti-RPRM antibody (SAB1102454, Sigma-Aldrich Chemie GmbH) was aligned against zebrafish Rprma and Rprmb using the same strategy described above.

### Zebrafish maintenance and husbandry

Wild-type (TAB5) zebrafish (*Danio rerio*) were maintained according to standard methods (Westerfield, 1995). Embryos were raised in system water at 28°C and staged according to hours post-fertilization (hpf) and morphological criteria (Kimmel et al., 1995). All zebrafish studies were conducted under the guidance and approval of the Institutional Animal Care and Use Committee and the Bioethical Committee at Pontificia Universidad Católica de Chile.

### cRNA probe synthesis and whole-mount *in situ* hybridization (WISH)

Templates for probe synthesis were PCR amplified from embryonic zebrafish cDNA using primers containing T7 RNA polymerase promoter sequence. To minimize cross-reactivity, the 5′ untranslated (5′-UTR) regions of *RPRM* genes were used for primer design. Primer sets were designed as follows: *rprma*, fw: 5′- TGAGGAGAACCTCCTGTGCT-3′, rv: 5′-TAA TACGACTCACTATAGGGGCCTGATCCTGATGGTTCGT-3′; *rprmb*, fw: 5′- TCCACCCATTCATCCTGTCA-3′, rv: 5′-TAATACGACTCACTATAGGGTCGGAGTTTCTTCGTTTGTG-3′; and *rprml*, fw: 5-GACCGGAGATCATCCAAAGA-3′, rv: 5′-TAATACGACTCACTATAGGGCTCGTTTCGTAAACGTGCAA-3′. All PCR products were of the expected size as inspected by agarose gel electrophoresis (data not shown). Purified PCR products were *in vitro* transcribed and labeled using digoxigenin (DIG) RNA labeling Kit (Roche) according to manufacturer's protocol. cRNA probes were purified using mini Quick Spin RNA Columns (Roche) and stored at −80°C with deionized formamide. WISH was carried out as described previously (Amigo et al., 2009). Embryos older than 24 hpf were treated with 0.003% 1-phenyl 2-thiourea (Sigma) to inhibit pigmentation.

### Immunohistochemistry (IHC) staining

Zebrafish embryos at 24, 48, and 72 hpf were fixed in 4% paraformaldehyde or Trichloroacetic acid (TCA -depending of developmental stage-) 2% overnight at 4°C. Fixed embryos were washed in PBT (1% Triton x-100) and subsequently treated with acetone for 20 min at −20°C, washed and consecutively treated with either Proteinase K (10 ng/mL; 24–48 hpf embryos) or trypsin 1x (72 hpf embryos). Blocking was carried out with 10% FBS + 1% DMSO in PBT twice for 1 h each and treated overnight with 0.1% H_2_O_2_. Embryos were then washed in PBT and incubated with primary antibody at 4°C for 3 days with mild shaking. Antibody dilution was prepared in blocking solution containing 0.2% (w/v) of blocking reagent (Roche). Rabbit polyclonal anti-RPRM antibody (SAB1102454, Sigma-Aldrich Chemie GmbH) and mouse monoclonal anti-acetylated tubulin (T6793, Sigma-Aldrich Chemie GmbH) were used in 1:800 and 1:1000 dilutions respectively; rabbit policlonal anti-GFP (A11122, Thermo Fisher Scientific) was used in 1:500 dilution. For secondary antibody labeling, goat anti-rabbit IgG Alexa-488 and goat anti-mouse IgG Alexa-546 (Invitrogen) were used at 1:200 dilution in blocking buffer for 2 h at room temperature in combination with Hoechst 33342 nuclear marker at 1 μg/mL (ThermoFisher Scientific Inc.).

### Knockdown of RPRM by morpholino (MO) microinjections

Embryos were microinjected at 1–2 cell-stage with 3 nL of morpholino solution (along with phenol red) at a concentration of 1 mM for *RPRM (rprma*: 5′-AGTCCAAGGCTTCAGACAGTGTCGT-3′ and *rprmb:* 5′-AATTCATGCTGAACTGCTGTTCTCT-3′) and 0.3 mM for standard control morpholino (5′-CCTCTTACCTCAGTTACAATTTATA-3′). *RPRM*-MOs were obtained from Gene Tools (Gene Tools, LLC) and designed to block the translation start site. Reduction in protein synthesis was confirmed by immunohistochemistry (IHC) when compared *RPRM*-MO injected embryos with controls.

### Imaging

After WISH, zebrafish embryos were embedded in 75% glycerol/PBS and imaged using NIKON eclipse 80i microscope equipped with a DS-Vi1 (NIKON) camera. For fluorescent whole mount immunohistochemistry, embryos were mounted in acrylic rings with glass bottoms containing 1% low melting point agarose and images were acquired using Leica TCS LSI macro zoom confocal microscope.

## Results

### Expression patterns of *rprm* (*rprma/rprmb*) and *rprml* during zebrafish neural development

**Figure 1:**
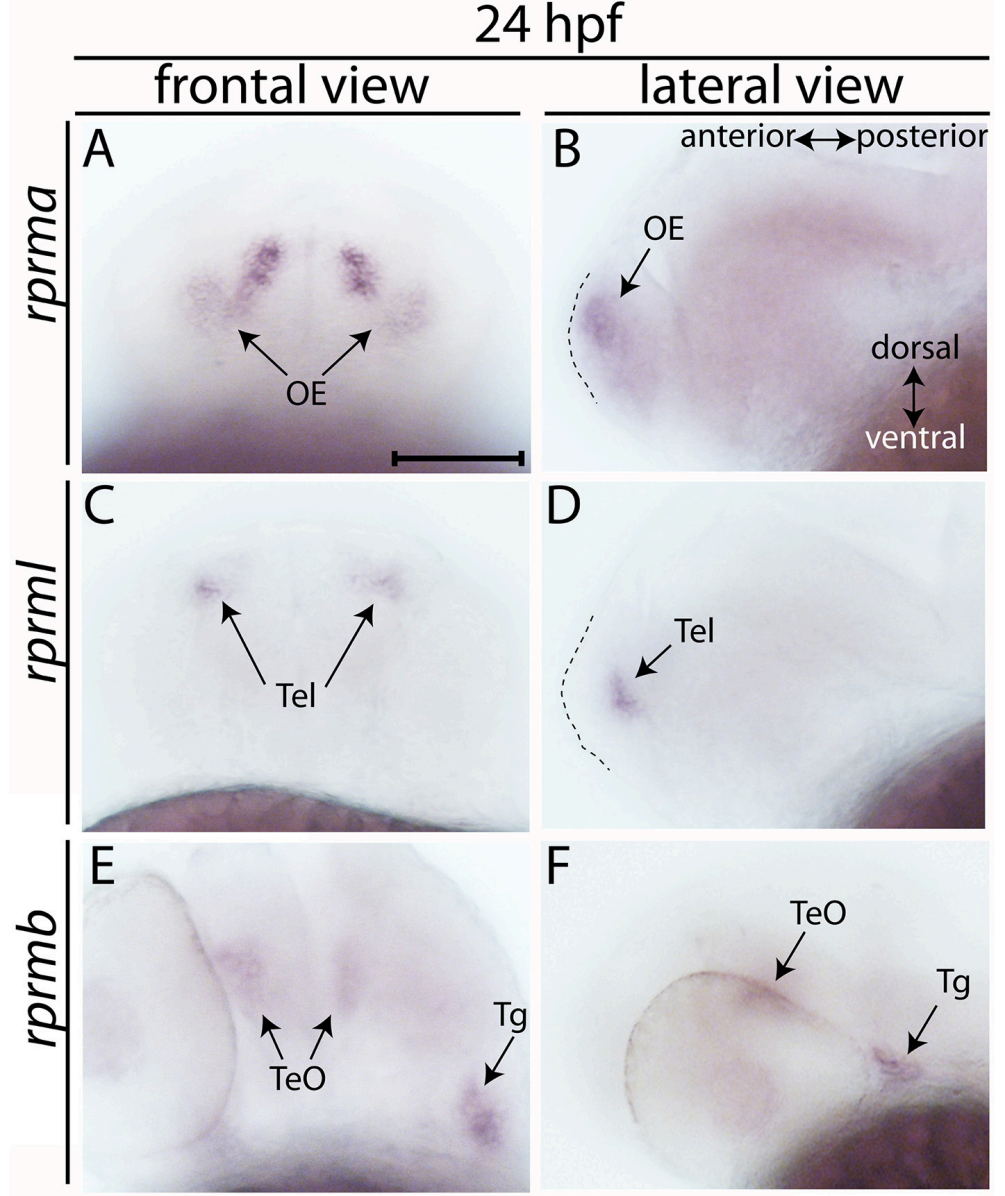
Expression of *RPRM* mRNA at 24 h post-fertilization (hpf). **(A–E)** The expression patterns of *rprma/b and rprml* were visualized by whole-mount *in situ* hybridization (WISH) during zebrafish early neuronal development. **(A,C,E)** Frontal views of the embryos heads, with dorsal to the top and ventral to the bottom (double arrow in **B**). **(B,D,F)** Lateral views of the embryos head with anterior to the left and posterior to the right (double arrow in **B**). **(B,D)** dashed areas represent the most anterior part of the embryo's head. At 24 hpf **(A,B)** *rprma*, **(C,D)** *rprml*, and **(E,F)** *rprmb* transcripts are located in neuronal cell populations such as: **(A,B)** olfactory placode (OP, black arrows), **(C,D)** telencephalon (Tel, black arrows), and **(E,F)** tectum opticum (TeO) and trigeminal ganglia (Tg), respectively. Scale bar in **(A)**: 100 μm.

**Figure 2:**
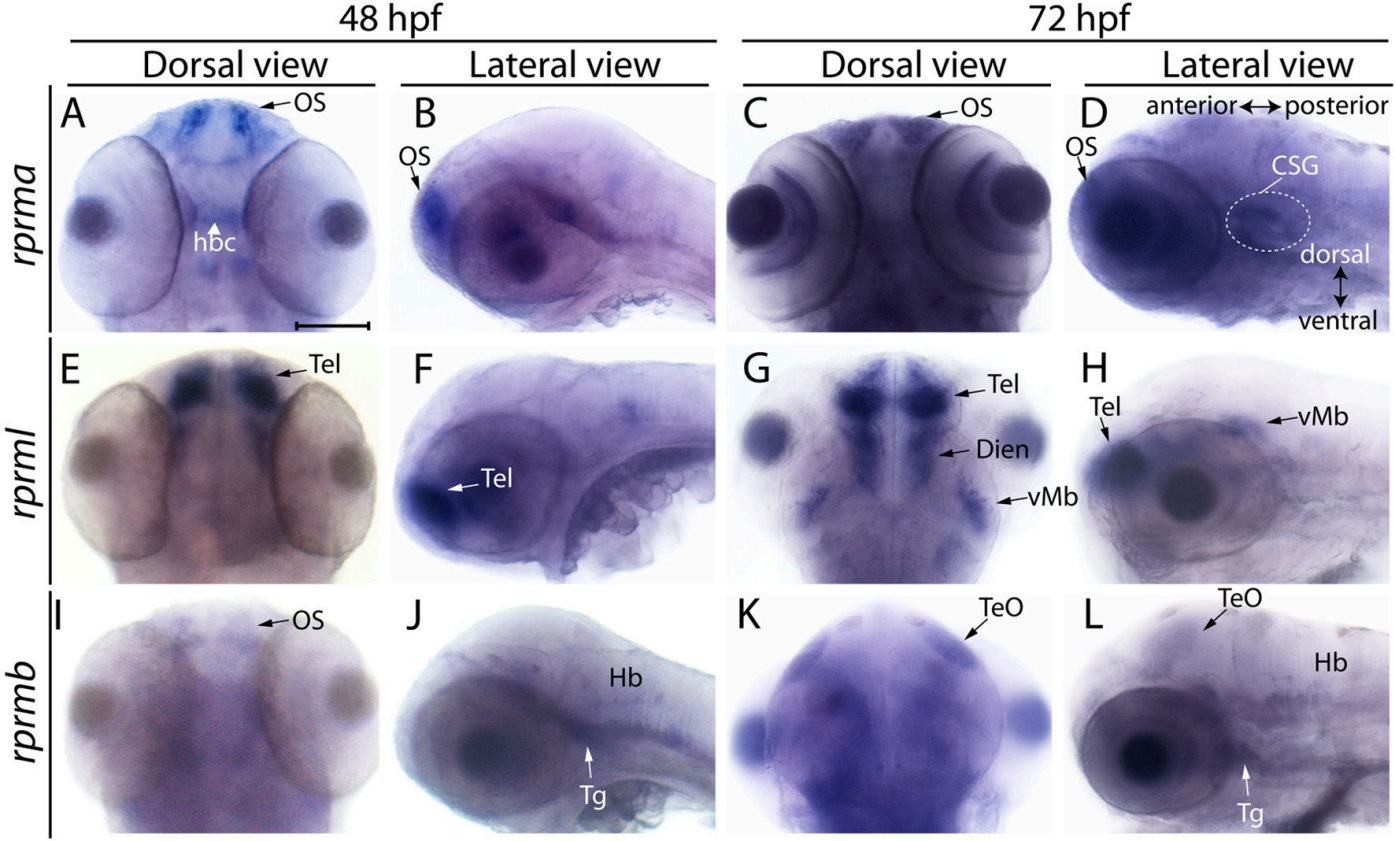
Expression of *RPRM* mRNA at 48 and 72 hpf in embryonic zebrafish. Expression of *RPRM* transcripts was examined using WISH in wild-type embryos. At 48 and 72 hpf, **(A–D)** *rprma*, **(E–H)** *rprml* and **(I–L)** *rprmb* transcripts are located in specific cell populations. **(A,C,E,G,I,K)** Dorsal views of the embryos head, with anterior to the top and posterior to the bottom. **(B,D,F,H,J,L)** Lateral views of the embryos head with dorsal to the top, ventral to the bottom (double arrow in **D**), anterior to the left and posterior to the right (double arrow in **D**). *rprma* is expressed in the **(A–D)** olfactory system (OS, black arrows), habenular commissure (hbc, white head arrow) and at 72 hpf in presumptive distal cranial sensory ganglia (CSG, doted circle in **D**). *rprml* is expressed in the **(E–H)** Tel (black and white arrows), **(G)** diencephalon (Dien) and **(H)** latero-ventral midbrain (vMb). *rprmb* is expressed in the **(I)** OS (black arrow), **(J)** Hb, trigeminal ganglion (Tg, **J**,**L**) and **(K,L)** TeO (black arrows). Scale bar in **(A)**: 100 μm.

Expression profiles of *rprma, rprmb* and *rprml* in the head region of embryos were analyzed by WISH from 24 until 72 h post-fertilization (hpf) (Figures 1, 2). At 24 hpf, *rprma* is expressed in the anterior surface of the forebrain (FB) corresponding to the olfactory placodes (OP) (Figures 1A,B). At the same developmental stage, expression of *rprml* is detected in the telencephalon (Tel) (Figures 1C,D), whereas *rprmb* is expressed in the tectum opticum (TeO) and the trigeminal ganglia (Tg) with no apparent expression along the FB/Tel (Figures 1E,F).

Between 48 and 72 hpf, expression of *rprm*a remains in the olfactory system (OS, Figures 2A–D), habenular commissure (hbc) (Figure 2A) and presumptive cranial sensory ganglions (CSG) (Figure 2D). While *rprml* mRNA expression was evident in the forebrain, telencephalon and diencephalon and at 72 hpf in the latero-ventral midbrain (vMb) (Figures 2E–H). At 48 hpf, weak *rprmb* expression was detected in the OS, Tg and the hindbrain (Figures 2I,J). At 72 hpf, *rprmb* transcript is now located at the TeO and Tg (Figures 2K,L). (Figueroa et al., 2017). The spatiotemporal expression patterns for *rprma, rprmb* and *rprml* indicate that these genes are expressed differentially during zebrafish neural development (Figures 1, 2).

### RPRM protein is expressed in the OP and OE of zebrafish embryos

**Figure 3:**
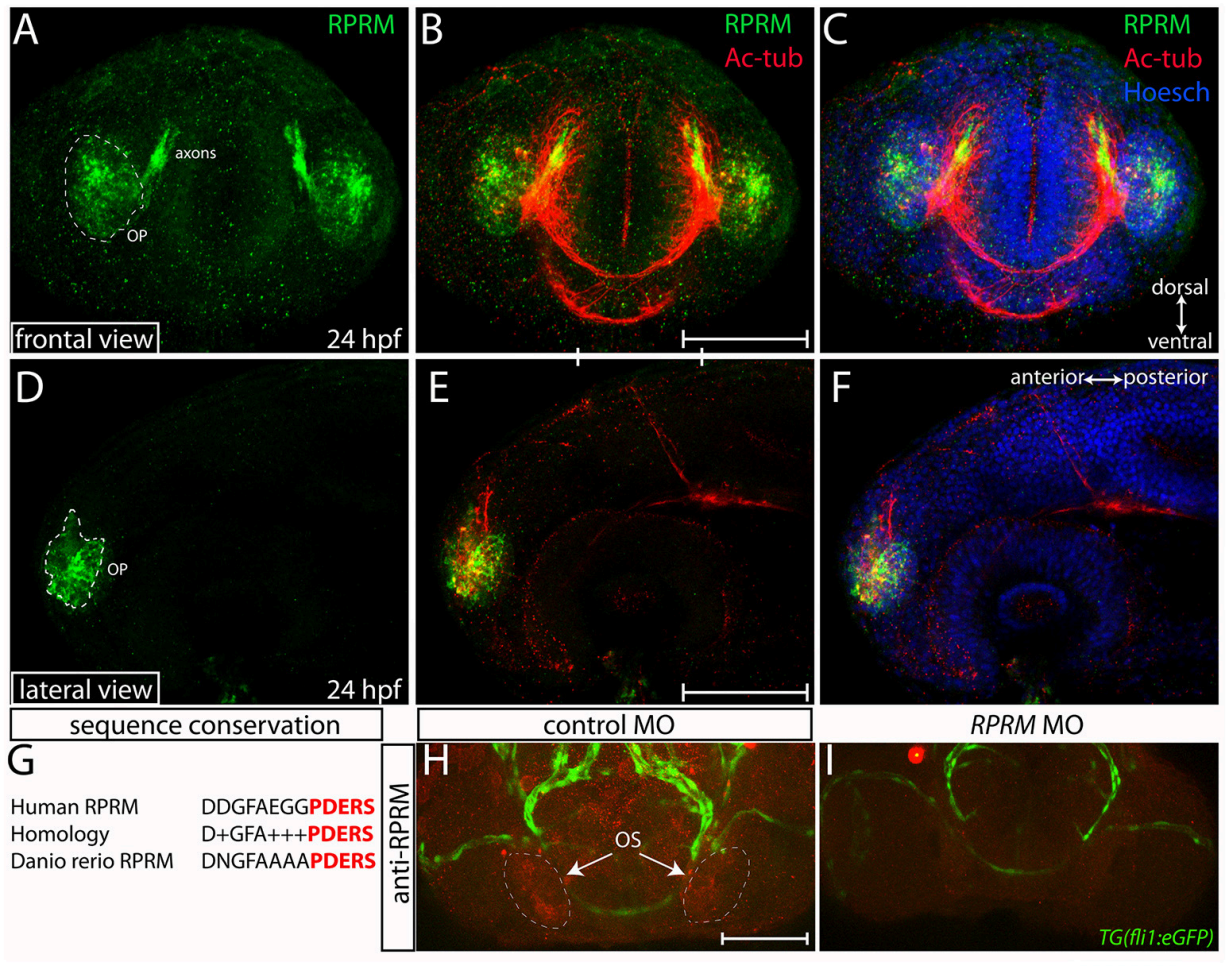
Expression of RPRM protein in the zebrafish olfactory system. RPRM protein localization was examined by immunofluorescence (IF) in wild-type embryos. At 24 h post-fertilization (hpf). **(A–C)** Frontal and **(D–F)** lateral views with dorsal to the top and ventral to the bottom (double arrows in **C**). **(A,D)** RPRM is expressed in the OP. The OP give rise to primary sensory neurons, and support basal cells of the OE. **(B,E)** Overlapped expression of RPRM with acetylated tubulin (Ac-tub) in the axons projecting to the presumptive olfactory bulb (OB) in the central nervous system (CNS). **(C,F)** Nuclei of the head cells are labeled by Hoechst staining. **(G)** Sequence conservation between human immunogen and zebrafish RPRM proteins. **(H,I)** RPRM protein expression is effectively blocked by antisense oligonucleotide MOs. **(H,I)** Frontal views of the head region in control-MO and *RPRM* MO-injected embryos by double immunofluorescence confocal microscopy at 48 hpf (with antibodies against RPRM and GFP). RPRM antibody labeled the olfactory system (OS, in red) in **(H)** MO-control injected embryos, but not in **(I)** *RPRM* MO-injected embryos. Scale bars in (**B,E,H)**: 100 μm.

Our study, together with previous evidence (Figures 1, 2 and Figueroa et al., 2017), show that the *rprma* transcript is prominently expressed in the PNS at the OP/OS. To investigate whether RPRM proteins are expressed in different subsets of cells within the peripheral and central nervous system (PNS/CNS), we performed IF coupled with confocal microscopy using a rabbit polyclonal anti-human RPRM antibody, which was generated against an immunogenic 13 amino acidic sequence that is partly conserved between zebrafish and human (Figure 3). By 24 hpf, RPRM is observed in the OP, a restricted domain in the anterior portion of the head (Figures 3A–F). At this developmental stage, RPRM protein also partially overlaps with the axonal marker anti-acetylated tubulin (Ac-tub) in axons projecting from the OP to the developing olfactory bulb (OB) (Figures 3B–C).

### Validation of anti-RPRM antibody in zebrafish embryos

An alignment of the human and zebrafish RPRM protein sequences revealed a striking conservation pattern on the predicted transmembrane and C-terminus domains of the proteins (Figure 3G), from where the immunogenic sequence comes. This information suggested that anti-human RPRM antibody may also work on zebrafish RPRM proteins. To validate the specificity of the RPRM antibody we performed knockdown of *RPRM* genes by using antisense oligonucleotide morpholinos (MOs). Reduction in immunoreactivity was confirmed by IHC in MO-injected embryos compared with control MOs (Figures 3H,I). Loss-of-function was performed in the transgenic *Tg(fli1:GFP)* line, using the GFP signal as a counter label for immunoreactivity (Figures 3H,I).

**Figure 4:**
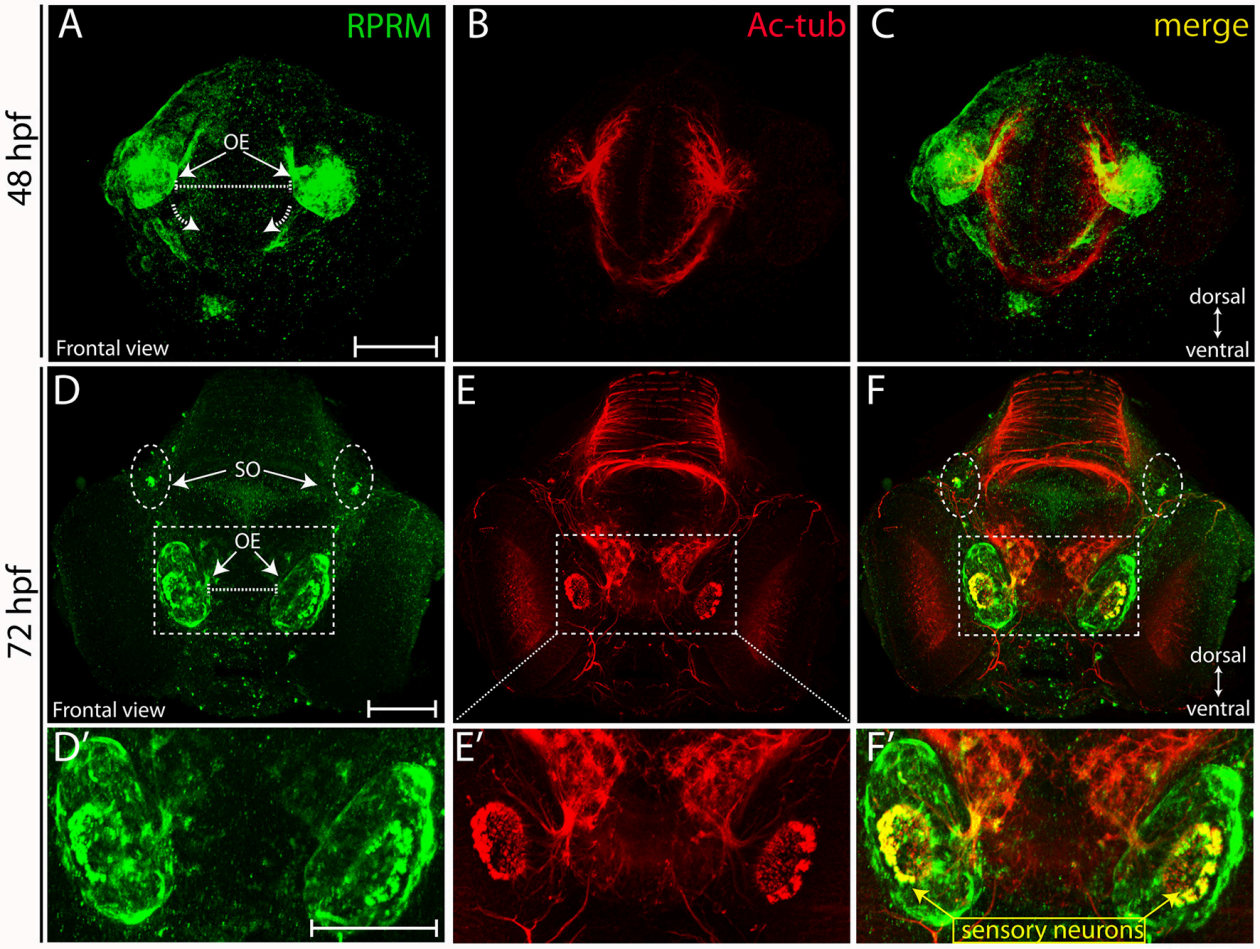
Expression of RPRM in zebrafish olfactory epithelium. RPRM protein expression was analyzed by IF in wild-type embryos. **(A–F)** Frontal views with dorsal to the top and ventral to the bottom (double arrows in **A,D**). At 48 hpf **(A–C)** RPRM is expressed in the OE showing co-localization with Ac-tub. **(B–C)**. At 72 hpf, **(D–F)** RPRM is expressed at the OE and co-localizes with olfactory sensory neurons (OSNs) which express Ac-tub **(F–F**′**)** and some of the axons projecting to the OB, also is present in the supraorbital neuromasts (SO) located bilaterally (dotted circles in **D, F**). **(D**′**–F**′**)** inset magnification at the OE showing RPRM and Ac-tub expression in OSNs (yellow square and arrows). Scale Bars in **(A,D,D**′**)**: 100 μm.

**Figure 5:**
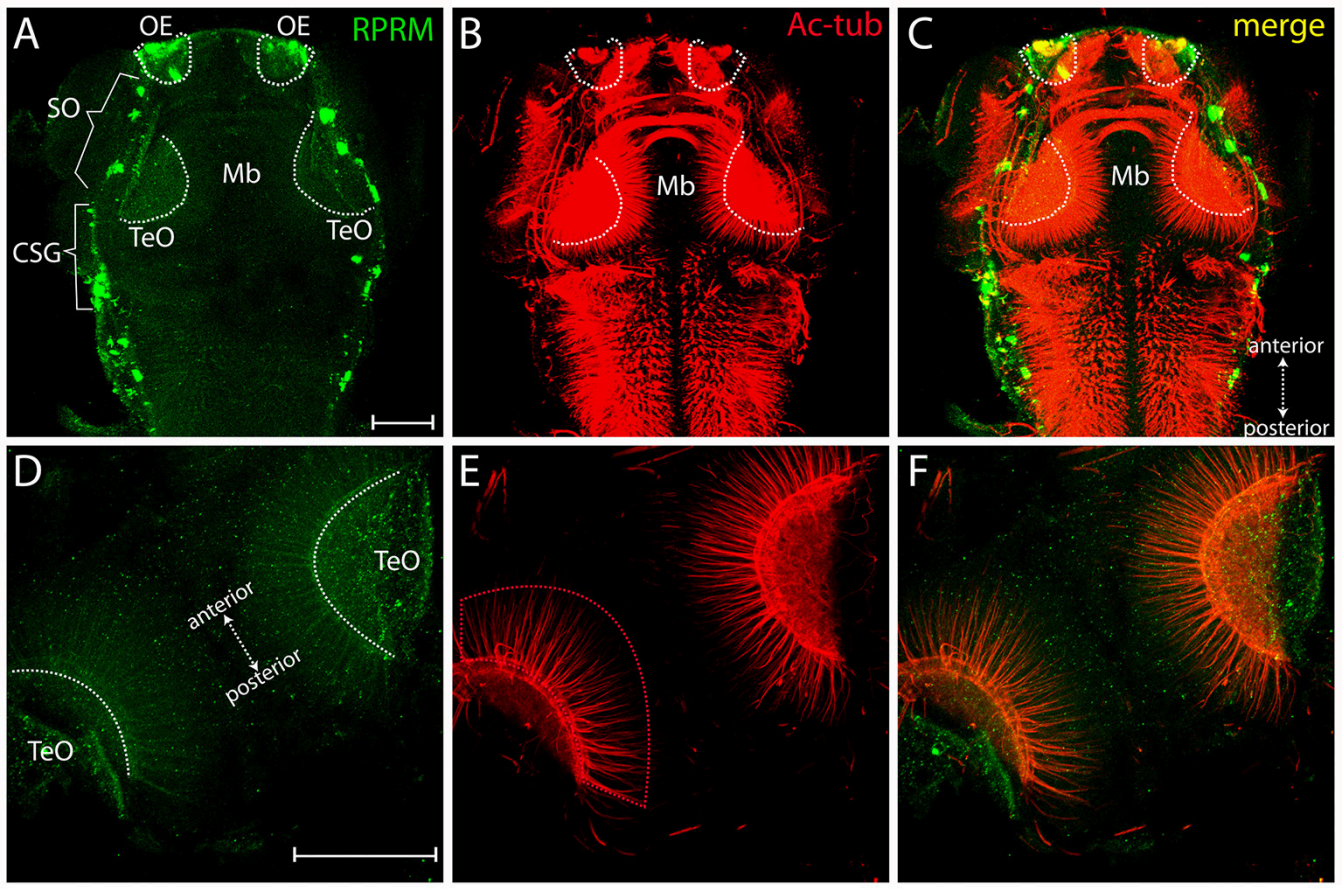
Expression of RRPM in zebrafish tectum opticum. Whole-mount immunofluorescence showing RPRM staining in a representative zebrafish embryo at 3dpf. **(A–C)** Dorsal view with anterior to the top, posterior to the bottom (double arrows in **C**). Expression of RPRM is shown in green and Ac-tub is shown in red. Positive labeling is observed in the OE, supraorbital neuromasts (SO, brackets), cranial sensory ganglia (CSG, brackets) and TeO (dotted white areas in **A–C**). **(D–F)** Magnification of TeO area with anterior to the upper-left and posterior to the bottom-right (double arrows in **D**), showing optic neuropil area labeled bilaterally (dotted white areas in **D**) as well as projections extending radially toward the midline (red dotted area in **E**). **(F)** Merged image for **(D,E)**. **(A–F)** All the panels represent z-stack projections. Scale Bars in **(A,D)**: 100 μm.

**Figure 6:**
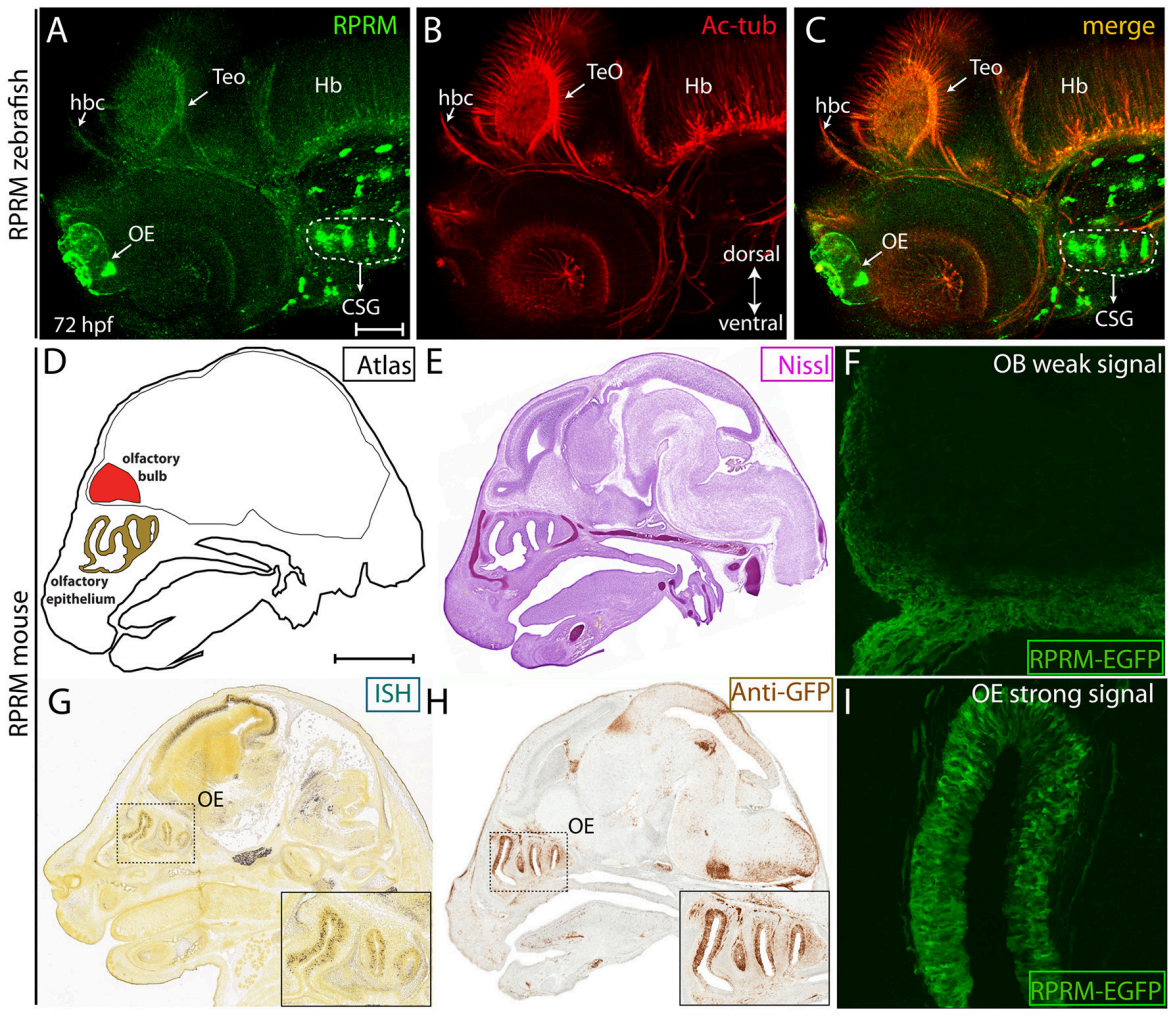
RPRM expression is conserved between zebrafish and mice. **(A–I)** Zebrafish RPRM protein expression, visualized by IF at 72 hpf, presents similar expression patterns when compared with murine RPRM in comparable developmental stages. **(A–C)** Lateral view of a representative 72 hpf embryo by IF, showing RPRM (green) and Ac-tub (red) expression in the habenular commissure (hbc), OE, TeO, CSG, and hindbrain (Hb). Dorsal is show to the top and ventral to the bottom (double arrow in **B**) **(D)** Sagittal atlas image of E15.5 mouse embryo showing reference areas of the OS (OE, OB). **(E)** Nissl staining of sagittal section from E15.5 mouse embryo. **(G,H)** *RPRM* expression as detected by *in situ* hybridization (ISH) and **(F)** immunohistochemistry (IHC) for anti-GFP in the transgenic mice line *TG(BAC-180MB-RPRM-EGFP)*. **(F–I)** *RPRM* expression pattern in *TG(BAC-180MB-RPRM-EGFP)* sections, showing low levels of *RPRM* gene products in the OB/OS **(F)** and high levels of expression in the OE **(I)**. Scale Bars in **(A)**: 100 μm, in **(D)**: 1 mm.

Over the following 48 h, RPRM expression maintain restricted to the anterior portion of the PNS (compared Figures 4A–D). At 72 hpf, the expression of RPRM is observed in cells adjacent to the apical surface, where the olfactory sensory neurons (OSN) are located (Figures 4F,F′, inset magnification), and the supraorbital neuromasts (SO, Figures 4D,F, 5A), distal cranial sensory ganglia (CSG) and the TeO (Figures 5A–C), showing positive staining in the optic neuropil (Figures 5D–6F), and the axons which project toward the stratum periventriculare (Figures 5E,F). At this developmental stage, RPRM is also located in the habenular commissure (hbc) and in the axons within the hindbrain (Hb) (Figures 6A–C). Our results indicate that in embryonic zebrafish, RPRM is prominently expressed in sensory organs such as the olfactory, optic systems, and ganglia originated from the epibrancheal placodes (Figures 5A–C,6A–C).

### Expression of *RPRM* in the olfactory system is conserved between zebrafish and mouse embryos

To test whether expression of RPRM is conserved between zebrafish and mice, we assessed RPRM protein expression in zebrafish at 72 hpf (Figures 6A–C) and mouse *RPRM* transcript (Figure 6E), and RPRM protein (Figure 6F) at E15.5 developmental stages. These two developmental stages are equivalent when comparing zebrafish and mouse developmental timeline (Kulkeaw and Sugiyama, 2012). In mouse, *RPRM* mRNA expression was determined by *in situ* hybridization (ISH) (available data from Allen Brain Atlas, http://mouse.brain-map.org) (Figure 6E) and *RPRM* gene products were analyzed in the mouse transgenic line *TG(BAC-180MB-RPRM-EGFP)* derived from a BAC clone (available data from GENSTAT, www.gensat.org; Figures 6F–I). Consistent with our findings in zebrafish, *RPRM* mRNA is clearly expressed in the OE in mice (E15.5, Figure 6E). Furthermore, enhanced green fluorescent protein (EGFP) expressed under the control of *RPRM* cis-regulatory modules showed a highly specific and strong EGFP signal in the Mb and OE (Figures 6G,I), while a weak signal was detected in the OB (Figure 6H). Of note, the EGFP expression is not necessarily a read out for RPRM protein expression. Since EGFP remains expressed even when RPRM is not. Nevertheless, given that this study looks a developmental stages, it is highly likely that the EGFP expression corresponds to the actual protein expression of RPRM. Importantly, the data from the transgenic mouse seems to overlap with the ISH data from the Allen Brian Atlas. Collectively, our findings indicate that expression of *RPRM* has been conserved throughout the evolution of the vertebrate nervous system.

## Discussion

In order to examine the roles of *RPRM* genes during the development of the nervous system we performed a detailed neuronal *RPRM* expression profile in zebrafish and mice. In both species, *RPRM* transcripts exhibit unique, although partially overlapping localization during neuronal development. Most importantly, the expression patterns for *RPRM* transcriptional/translational products are highly conserved in both species, as demonstrated by IHC/IF, WISH and transgenic fluorescent reporters. Together, our findings indicate that *RPRM* genes might possess a pivotal role during neuronal development and that the transcript and protein expression have been conserved during the evolutionary history of vertebrates.

### Distinct expression patterns characterize *RPRM* gene products in the developing nervous system

Each *RPRM* gene*, rprma, rprmb* and *rprml*, has very specific expression patterns during neuronal development in zebrafish embryos. The expression of *rprma* and *rprml* are largely restricted to defined regions such as OE and Tel, respectively. Conversely, *rprmb* transcript is expressed in most posterior neuronal territories such as TeO (dorsal mesencephalon) and Tg. The observed expression of *rprmb* mRNA in the TeO is consistent with RPRM protein expression, detected by IHC/IF, in the zebrafish tectal region (Figures 5A, 6A). Importantly, *rprma* is the only *RPRM* gene expressed in the OP and OE, suggesting a putative role for *rprma* during the development of the olfactory system. In summary, three zebrafish *RPRM* genes (*rprma, rprmb*, and *rprml*) are expressed in different regions of the embryonic PNS and CNS, which indicate a process of subfunctionalization during the evolutionary history of this group of genes and a specific role for each *RPRM* gene during nervous system development.

### *RPRM* expression in the olfactory system

In vertebrates, the PNS arises fundamentally by tissue-interactions between the neural crest and sensorial placodes (Steventon et al., 2014). In the zebrafish, the OPs appear as thickenings at the anterior ectoderm by 17–18 hpf and these thickenings later invaginate to form the OE covering the opening of the nose, by 32 hpf (Hansen and Zeiske, 1993). Here we show that Rprm protein is located in the OP and that expression in this territory is maintained throughout specification of the OE (Figures 3, 4). At 24 hpf, a unique subset of pioneer axons projects caudally from the OP to form the olfactory nerve and establishes glomerular-like structures in the presumptive OB (Whitlock and Westerfield, 1998). The axons we have detected using an antibody against RPRM seem to be pioneer axons (Figure 3), as no other projections exit from the OP at this developmental stage. Interestingly, the OE is one of the few tissues that present neurogenesis during adulthood due to the persistence of a pool of embryonic neuronal stem cells (NSCs) (Madelaine et al., 2011). Previously, *RPRM* has been described as regulator of cell cycle (Ohki et al., 2000), thus *RPRM* could potentially play a role during self-renewal of NSC in the adult olfactory system.

### Comparison of *RPRM* gene expression patterns between zebrafish and mice

Our previous studies in human revealed that RPRM protein was expressed in the brain (Figueroa et al., 2017). Moreover, mice embryos showed strong *RPRM* transcriptional expression in the OE, using ISH and/or the mouse reporter transgenic line *TG(BAC-180MB-RPRM-EGFP)*. This expression pattern is consistent with the mRNA and protein localization observed in the olfactory system of zebrafish embryos (Figure 6). In mice, *RPRM* is broadly expressed in the habenular nuclei, Mb and Hb; in accordance with *RPRM* expression pattern in embryonic zebrafish. Altogether, our findings indicate that the conserved expression of the *RPRM* gene family through vertebrates might implicate an essential role for *RPRM* during neural development. Future mechanistic studies will be required to clarify *RPRM* function in neuronal tissues.

### Evolutionary role of the *RPRM* gene family

*Reprimo* genes seem to be an evolutionary innovation of vertebrates as no traces of them have been annotated in other taxa (Wichmann et al., 2016). This group of genes diversified as a consequence of the two rounds of whole genome duplications occurred early in the evolutionary history of vertebrates (Wichmann et al., 2016). In teleost fish (e.g., zebrafish, *Danio rerio)* the repertoire of *RPRM* genes further expanded as a consequence of the teleost-specific genome duplication (Meyer and Van de Peer, 2005; Kasahara, 2007). Thus, current species of teleost fish possess a repertoire of four reprimo genes: *rprma, rprmb, rprml*, and *rprm3* (Wichmann et al., 2016). Transcriptome studies in representative species of vertebrates have shown that *RPRM* genes are mostly expressed in the CNS, and that this pattern has been maintained for more than 500 million years during the evolutionary history of the group (Wichmann et al., 2016). Although *RPRM* genes have a function during the cell cycle and the neurons in general does not experience cell division, their expression in the adult nervous tissues could be seen as a protective measure within this organ (Zohrabian et al., 2007). More recently, we determined the *RPRM* expression profiles during embryonic development using zebrafish as a model system (Figueroa et al., 2017). In agreement with our *in silico* analysis, *RPRM* genes are expressed in the developing brain but also in digestive tube and blood vessels (Figueroa et al., 2017). In the present study we found that within the PNS, *RPRM* is mainly expressed in the OE and that this pattern is shared with mammals. The similarity in the *RPRM* expression pattern between both groups is consistent with the fact that at the anatomical level the general organization of the olfactory system is conserved among vertebrates (Kermen et al., 2013; Saraiva et al., 2015). The olfactory epithelium (OE) of bony fish, where reprimo genes are mostly expressed, is also conserved among vertebrates.

At the amino acid level, there is a striking conservation throughout the C-terminus of the *RPRM* family, suggesting a strong selective pressure of this domain that could be related to the functional role of this protein family. The conservation of the amino acidic sequences between zebrafish and human *RPRM* proteins, as well as the conservation of the expression pattern of these genes, suggest that *RPRM* proteins might play similar roles in both groups.

Due to the highly specific and conserved *RPRM* expression in the brain and specific sensory organs in different vertebrates, *RPRM* genes might play a pivotal role during sensory neurons development, such as olfactory receptors as well as other detected sensory system structures like SO and CSG, all originating from neural crest and ectodermal placodes. With our present study, we provide novel knowledge for future investigation directed to determine the physiological functions of *RPRM* in vertebrate PNS/CNS, from the cellular level to animal behavior.

## Author contributions

KS and AQ: conceptualization, data curation, formal analysis, validation, methodology, writing (editing); CL: data curation; IW, AC, and GO: writing (editing); JO: conceptualization, formal analysis, writing (editing); MC: conceptualization, formal analysis, writing (editing), funding acquisition. JA: conceptualization, data curation, formal analysis, validation, methodology, funding acquisition, project administration, supersision, writing (editing), writing (editing, original draft).

### Conflict of interest statement

The authors declare that the research was conducted in the absence of any commercial or financial relationships that could be construed as a potential conflict of interest.
